# Extracts from the Records of the Boston Society for Medical Improvement

**Published:** 1856-09

**Authors:** 


### *Extracts from the Records of the Boston Society for Medical Improvement.*

By F. E. Oliver, M. D., Secretary.

June 9th. *Glandular Hypertrophy of the Breast, of twenty-eight years' Duration.* Dr. Clark showed the specimen and reported the case.

S. F., a married lady, 62 years of age, presented herself at the Massachusetts General Hospital with a very large, movable tumor of the right breast, of which she gave the following history: Twenty-eight years since, she noticed a small, hard lump in the right breast, which did not alarm her, as it was neither painful nor tender. Soon after this she nursed a child, and from that time she thinks its growth commenced. At first of the size of a common bean, it soon became as large as a hen's egg, and gradually increased till last year, since which time its growth has been very rapid. It now occupied the whole gland, and was at least three times its natural size. The axillary glands were not affected, and her general health was good.

The disease was considered to be *glandular hypertrophy*, and the removal of the tumor was recommended.

The next day it was removed by the usual elliptical incisions. The flaps of skin came readily together, and were secured by sutures and adhesives.

June 12th, the fifth day, erysipelas, in a mild form, made its appearance about the wound, and gradually extended, during the next ten days, over the whole chest, down the back and over the right arm. During the next ten days it gradually subsided and disappeared. The patient was discharged well, July 11th.

There was but little constitutional disturbance during its whole progress.

The treatment consisted of the application of dried flour locally, and the administration of quinine internally, with nourishing diet, ale and cider *pro re nata.*

The tumor weighed two and a quarter pounds; was lobulated, and involved the whole breast, extending to the oxilla. In its centre was found a rounded, calcareous mass, resembling in shape and size the head of the humerus.

The following is Dr. Shaw's statement of the microscopical appearance of the portion examined by him:

"The fragment of tumor appears to be a glandular hypertrophy.

"It is composed of lobules in which the epithelium is well marked, with abundant fibrous tissue, and a quantity of fibro-plastic elements, such as are commonly found in hypertrophical glands. There is no cancer structure apparent under the microscope."

Dr. Ellis gave the following as the result of his examination:

"Fragment of tumor contained much fibrous tissue and many lobules, which looked like masses of small nuclei. In the latter no nucleoli could be distinguished. Numerous small, round or oval free nuclei were, however, seen, containing one or two small nucleoli. Tubuli, lined with epithelium, also were noticed. The disease was evidently glandular hypertrophy."

He adds:

"I examined it also with reference to the existence of cysts. On puncturing many parts of the external surface, there exuded a somewhat milky fluid, which had been contained in fissures or flattened cavities of no great extent. The fluid contained numerous cells and free nuclei of an epithelial character. Projecting into the fissures from various parts of their walls, were warty vegetations, some of which had a linear or laminated arrangement. One of the largest of these cavities contained a yellowish fluid, somewhat resembling olive oil. In this, in addition to the epithelium, were found many fat globules, and large spherical corpuscles, apparently formed by the aggregation of the same."

June 9th. *Mammary Fibro-plastic Tumors.* Case reported by Dr. Gay.

In connection with the case mentioned by Dr. Clarke, Dr. Gay referred to that of Ellen McFaine, 25 years old, who entered the hospital, August 29, 1855, with a similar disease of the right breast.

The patient could refer to nothing, as an assignable cause of the disease, but a pretty severe blow in that region, from her husband, three years before. A temporary suffering followed, and in a very short time the part resumed, apparently, its natural position.

A year ago, for the first time, she noticed a small, movable "bunch," of the size of a hazel nut, situated to the inside of the right nipple, midway between that and the sternum. There was no change in the color of the skin, nor the slightest pain. Its growth had been very slow till within the last three months. Six months after she first perceived it, it was of the size of a hen's egg. For the last three months it had enlarged very rapidly, the patient saying, that it seemed to her as if she could almost see it grow. For the last eight months, the pain had been exceedingly severe, of a darting, leaping character, and always in one and the same spot, the place of origin of the disease. The color of the skin remained natural till three months ago, when the subcutaneous veins became enlarged and tortuous. Soon a capillary redness of the skin was seen, which slowly increased. At present, there is a large irregular swelling with firm lobes, elastic, not painful on pressure, freely movable and pendulous, inconvenient from its weight and dragging; the circumference about its base being 19¾ inches, and from skin to muscle, 4½ inches. The skin was nowhere adherent to the tumor.

Sept. 1st, the tumor was removed, with a large portion of skin, including the nipple. There was no unusual bleeding, but a few small vessels requiring ligatures. Its weight was two pounds eight ounces. Under the microscope, fibro-plastic material was found in large quantities, together with what might be called glandular hypertrophy. The wound healed kindly, and the patient was discharged well, on the 25th of the same month.

The patient continued very comfortable, and without any trouble, till some time in the following December, when she noticed a small growth, near the old cicatrix, of the size of a pea, and perfectly movable. This was very rapid in its growth, much more so than the first, and attended with severe lancinating pains. When she entered the hospital the second time, May 22d, 1856, the tumor was about twice the size of the former one, with no difference, except in size, in the symptoms, feel, and general appearance. There seemed to be three distinct large lobes, with some adhesion to the skin, in the neighborhood of the cicatrix. Its deep attachments were very loose, so that it was freely moved in every direction. it extended higher up into the axilla, much more so than in September. There was no enlarged axillary or cervical gland. The patient was anxious for an operation.

May 24th. The tumor and cicatrix were removed without the least difficulty. The lips of the wound were not brought together, as it was desirable to have it closed by granulations. On a section of the mass, it presented the same appearances to the eye, exhibited by the first tumor. It weighed 4¼ lbs. Under the microscope, a precisely similar structure was found, namely, fibro-plastic material, and glandular hypertrophy.

This case is interesting, from its rapid recurrence, about three months from the first operation, its rapid and great growth in about five months, and the lancinating, leaping character of the pain. The recurrence and increase were as rapid as is seen in cancerous affections. The same lancinating character of the pain was present as in scirrhous masses.

Another important and interresting case, alluded to by Dr. Gay, was that of Miss Cushing, of Hull, aged 40 years. She had observed an increase in the size and hardness of her left breast, for several months, without any particular reason. At no time had there been any pain. As it had enlarged very rapidly, the week previous to the operation, she became alarmed, and advised its removal. Accordingly, on the 3d of April, 1855, a large, regular, perfectly movable tumor of the left breast, from four to five inches in diameter, and four inches thick from skin to pectoral muscle, was removed. The skin was perfectly natural in color, softness and mobility. The wound healed in a short time, without any unpleasant symptom. There was a recurrence, and on June 8th a bosselated, elastic tumor, of the size of the first, principally involving the cicatrix, was again removed. July 25th, a tumor similar in size and general look, near the last cicatrix, was removed. Sept. 27th, another tumor, adherent at the base, was removed, together with a portion of the muscle. Oct. 12th, another operation was performed, removing every thing down to the intercostal muscle. Oct. 23d, the disease had returned in about the same spot, was strongly adherent, and as large as an orange. Another operation was not advised. There was no glandular enlargement. During the whole period of the progress of the disease, the patient had been almost entirely free from pain.

Under the microscope, the fibro-plastic material always preponderated. There was nothing like cancer cell.

The patient lingered till the 8th of January, 1856, when she died. At the time of her death, the tumor was enormous, being in circumference as large as a large wash-bowl, and five inches or more in thickness, reaching up to the patient's chin. From the attending physician's account, it must have weighed ten or fifteen pounds.

Here is the recurrence of a disease, and rapidity of progress, unquestionably much greater, in the same period of time, than in any form of cancerous growth.

At each time the operation was urged by the patient. In ten months from the first operation, the disease terminated fatally—after five separate recurrences, and four operations at intervals of from one to two months.

Another case of fibro-plastic disease was referred to by Dr. Gay, where there was at least three different operations, then a further recurrence, which was fatal, within a year.

Also, still another case, at present of about a year's standing, in which a recurrence was noticed four or five months after an operation, and now any attempt at removal is perfectly hopeless.

Dr. Bigelow remarked upon the connection between sero-cystic disease, which seems to be comparatively common abroad, though not so here, glandular hypertrophy, and the form of disease presented in the case of Dr. Clark. The sero-cystic disease, consisting of serous cysts, which contain more or less hypertrophied glandular tissue hanging off into their interior, and perhaps at last filling their cavities, causing distension of the breast, rupture of the surface, and ulceration; the glandular hypertrophy, in its common form, presenting rounded, hard tumors, movable, and generally not recurrent; and being, together with the present form of disease, equally characterized under the microscope, by the lobulated and somewhat irregular glandular outline distinctive of hypertrophied gland. But the form of the disease which is now shown to the Society he believed to be of comparatively rare occurrence. It is the second case reported here, and the third specimen which has been shown to the Society. The first specimen was removed at the hospital by Dr. Hayward, some years since, and was then exhhibited to the Society by Dr. Bigelow. It again recurred, and the patient entered the hospital about two years after, when it was again removed by Dr. Warren. It is this recurrence which characterizes this variety of glandular hypertrophy, and which, indeed, is not its only affinity to malignant or semi-malignant growths; for while it presents, microscopically, the common appearance of hypertrophied glandular tissue, it also presents a very large proportion of simple cellular and nucleated tissue, destitute of glandular structure; this fibro-plastic tissue apparently increasing with recurrence of the growth, and, at last, greatly predominating over the glandular structure. The gross appearance of this disease differs from those previous described, in being, to all appearance, a simultaneous and uniform enlargement and transformation of the whole breast, instead of a cystic disease with internal growths, or a separate mass of hypertrophy, as in the tumors before alluded to.

In reply to an inquiry of Dr. Gay, how Dr. B. explained the recurrence of the disease after the complete removal of the breast, Dr. B. remarked, that when the very considerable size of these breasts, and the soft, and, in some places, gelatiniform character of the tissue is considered, together with its adherence to surrounding structures, the great difficulty of completely removing every portion of the diseased tissue is apparent. Dr. B. considered that in this case, as in the frequently occurring cases of soft tumors of a cellular structure elsewhere, not surrounded by a distinct and firm outline, small portions will inevitably be left in the dissection, in spite of every precaution; especially when it is impracticable, as here, to remove the adjoining healthy tissue, which has been exposed to infiltration by the growth. Dr. B. gave it as his impression, that the softer this tissue, the greater is its tendency to return.

Dr. Clark had seen two cases of the partial hypertrophy, before described, within the past year.

In referring to the case reported by Dr. Gay, Dr. Abbot inquired as to the expediency of operating, when there is such liability to recurrence, this evidently having been hastened in the case alluded to.

Dr. Gay stated in reply, that he operated, in the case referred to, at the urgent request of the patient.

Dr. Bigelow was of the opinion that the patient should have the benefit of the chance in such cases, especially as rapidly-increasing disease is the patient's only alternative; and as there is yet some doubt about the character of this disease, which is, as far as is now known, purely local.

Dr. Clark remarked that he had seen several cases of glandular hypertrophy where no recurrence took place, and thought that of Dr. Gay exceptional.

With regard to the use of words in such cases, Dr. Bigelow remarked that he did not call a tissue malignant so long as it presented glandular structure.

Dr. Gay stated that Dr. Ellis did not find any trace of glandular structure in the specimen from the case reported by him, which was after repeated recurrence and removal.—*Boston Journal.*

